# Exposure to the insecticide-treated bednet PermaNet 2.0 reduces the longevity of the wild African malaria vector *Anopheles funestus* but GSTe2-resistant mosquitoes live longer

**DOI:** 10.1371/journal.pone.0213949

**Published:** 2019-03-14

**Authors:** Ange Tchakounte, Magellan Tchouakui, Chiang Mu-Chun, Williams Tchapga, Edmond Kopia, Patrice Takam Soh, Flobert Njiokou, Jacob Miranda Riveron, Charles S. Wondji

**Affiliations:** 1 Centre for Research in Infectious Diseases (CRID), Yaoundé, Cameroon; 2 Research Unit LSTM/OCEAC, Yaoundé, Cameroon; 3 Department of Animal Biology and Physiology, Faculty of Science, University of Yaoundé, Yaoundé, Cameroon; 4 Department of Vector Biology, Liverpool School of Tropical Medicine, Pembroke Place, Liverpool, United Kingdom; 5 Department of Mathematics, Faculty of Science, University of Yaoundé, Yaoundé, Cameroon; Universita degli Studi di Camerino, ITALY

## Abstract

**Background:**

Despite the increased report of insecticide resistance in malaria vectors, its impact on mosquito’s life-traits after exposure to insecticide-treated nets remains under investigated. Here, we assessed the effects of exposure to PermaNet 2.0 on several life traits of *An*. *gambiae* s.l. and *An*. *funestus* s.l. field mosquitoes in Cameroon.

**Methodology:**

Female *Anopheles* mosquitoes were collected indoor using electric aspirators in southern Cameroon (Obout) in 2016. After assessing the resistance status of F_1_ from the field collected-mosquitoes, progeny of the first generation (*An*. *funestus* s.l.) and seventh generation (*An*. *gambiae* s.l.) were used to assess the long-term effect of exposure to PermaNet 2.0 on several life-traits of these vectors (longevity, blood feeding ability, fecundity and fertility) in comparison to untreated net. In addition, the L119F-GSTe2 mutation associated with DDT/pyrethroids resistance in *An*. *funestus* was genotyped to assess its association with increased life-span post-exposure.

**Principal findings:**

Both *An*. *funestus* and *An*. *gambiae* were resistant to pyrethroids and DDT with a greater level in the latter. Pyrethroid-only nets PermaNet 2.0 (17.5% mortality) and Olyset (0% mortality) exhibited a significantly reduced efficacy against *An*. *funestus* in contrast to a greater efficacy for PBO-based Nets Olyset Plus (65% mortality), PermaNet 3.0 top (100% mortality). In both species, mosquitoes that survived exposure to PermaNet 2.0 exhibited a significantly reduced longevity than those non-exposed (6.95 days vs 12.46 for *An*. *funestus P<0*.*001*; 8.87 vs 11.25 days for *An*. *gambiae; P<0*.*001*). However, no significant difference was observed for blood feeding and fecundity in both species. In addition, molecular analysis of the L119F-GSTe2 mutation revealed that this mutation is associated with an increase in the chance of surviving after exposure to this net in *An*. *funestus*.

**Conclusions:**

These results show that although the PermaNet 2.0 presents a reduced efficacy against resistant populations, it remains efficient after exposure by reducing the life expectancy of the vectors which could contribute in the reduction of malaria incidence.

## Introduction

Malaria remains one of the most important vector-borne diseases despite control efforts including insecticide-based interventions [1]. However, there was a significant decrease in malaria incidence and mortality between 2000 and 2015, with over 70% of success attributed to vector control mainly through indoor residual spraying (IRS) and Long-Lasting Impregnated Bed nets (LLINs) [2]. After a decade of massive use of this control tools, insecticide-resistance is considered as a serious threat to malaria control programs. This risk from insecticide resistance is further supported by the recent report from WHO that after nearly two decade of decrease, malaria cases have increased in 2016 and 2017 [3]. Furthermore, recent observations that LLIN incorporating the synergist piperonyl butoxide (PBO) was more efficient in reducing malaria transmission than pyrethroid-only nets support the risk posed by resistance to the efficacy of LLINs [4]. However, there is an ongoing debate about the extent to which resistance to insecticides is reducing the effectiveness of LLINs against resistant mosquitoes.

It remains very challenging to connect insecticide resistance with an increase in malaria transmission as it is an inherently difficult relationship to measure, since a very good entomological monitoring is needed with thorough disease surveillance [5]. Many confounding factors, such as environmental conditions, affect also malaria incidence contributing to its fluctuation year to year. Elucidating the impact of exposure to the insecticides on the life traits of the major malaria vectors can provide more information on the efficacy of control measures and contribute to design and implement suitable resistance management strategies. If most studies have focused on the immediate impact of LLINs on mosquitoes, notably immediate mortality populations [6, 7], less study has been done on the long-term effects of the interaction of mosquitoes with LLINs which are much likely to impact the role in malaria transmission of mosquitoes under control. However, a recent study [8] revealed that LLINs may curtail the vectorial capacity of mosquitoes in *An*. *gambiae* through reduced mortality in exposed mosquitoes. Indeed, a decline in survival was reported for two resistant laboratory strains of *An*. *gambiae* s.l. after exposure to PermaNet 2.0. Such sub-lethal effect of insecticide-treated nets can contribute in the reduction of malaria transmission as the vectors have to live long enough to be able to develop the parasite until the infective stage. However, this was done with lab strains and further evidences are needed with natural populations and other major vectors such as *An*. *funestus*. Furthermore, the long-term effect of this exposure to LLINs remains to be characterized on several life traits of mosquitoes likely to impact their role in malaria transmission including fecundity/fertility and blood feeding. Therefore, to help to better assess the impact of resistance on the overall effectiveness of LLINs, we evaluated the long-term effect of exposure to the commonly used LLINs PermaNet 2.0 against populations of both *An*. *funestus* and *An*. *gambiae* in a same location in southern Cameroon. This study revealed a significant effect of exposure to PermaNet 2.0 on the longevity and fertility while highlighting that a glutathione-S transferase mediated metabolic resistance (L119F-GSTe2) was increasing the ability to survive after exposure to PermaNet 2.0.

## Materials and methods

### Study site

Indoor resting female mosquitoes (F_0_) were collected in Obout, central Cameroon (3° 7'0 "N, 11° 65'0" E). Climate of this area is of Guinean equatorial type, characterized by four seasons [9]. The hydrography is dominated by the Mefou River and its tributaries. There are also several other aquatic environments, whether linked to man-made activities bordered by vegetation, which constitute potential breeding site of *An*. *funestus*. Most houses are made of temporary materials, without ceilings, with many spaces facilitating access to mosquitoes. Insecticide-treated mosquito nets widely distributed by the government are being used by more than 70 percent of the population against mosquito bites.

### Sampling method

Blood fed females and/or gravid mosquitoes of *An*. *funestus* were collected indoor in July 2016. No specific permissions were required for these study but only a verbal agreement from the district chief and the household owners before mosquito’s collection each morning. In addition, mosquitoes *An*. *gambiae* from the same locality taken in the seventh generation and collected the month of January 2015 were used considering the scarcity of this species during the collection period. Indoor resting mosquitoes were collected indoor using electric aspirators. Mosquitoes collected were placed in a cage covered with a wet towel and brought back to the LSTM/OCEAC laboratory. Eggs from field collected mosquitoes were obtained using a forced egg laying method described previously [10] and put in cardboard cups containing mineral water for hatching. Larvae were reared till adults and females of the new generations obtained were split into two groups (experimental and control).

### Species identification

All female F_0_ mosquitoes were identified morphologically as *An*. *funestus* and *An*. *gambiae* using a morphological identification key [11]. Genomic DNA was extracted from field-collected mosquitoes using the Livak protocol [12]. A PCR cocktail was performed for the specific identification of the species of the *An*. *funestus* group. [13] and the *An*. *gambiae* complex [14].

### Infection of malaria vectors by *Plasmodium* parasites

*Plasmodium* infection rate was estimated by Taqman assay using the head/thorax and the abdomen of F_0_ field-collected mosquitoes as previously described [15, 16]. Only females of *An*. *funestus* sensu stricto (s.s.) were used for this assessment since *An*. *gambiae* were not sufficiently present in the study site during the collection period.

### Insecticide susceptibility assays

Susceptibility to insecticides across all four major public health insecticide classes was tested in *An*. *funestus*: insecticides tested included the pyrethroids permethrin (0.75%), deltamethrin (0.05%), etofenprox (0.5%); the organochlorines DDT (4%), dieldrin (4%), the carbamates bendiocarb (0.1%), propoxur (0.1%) and the organophosphates malathion (5%) and fenitrothion (1%) (VCRU, Penang, MALAYSIA). For *An*. *gambiae*, susceptibility tests were performed only for DDT, permethrin and deltamethrin. Bioassays were performed on F_1_ mosquitoes aged 2–5 days using WHO susceptibility test kits and standard protocol [17] for F_1_ adults under ambient room temperature ranging from 25–28°C and relative humidity of 75–80%. Four replicates of 25 unfed mosquitoes each were used for the bioassay. Each replicate was exposed to the insecticide-impregnated filter paper for 60 minutes and the knockdown recorded before transferring them back into the holding tube supplied with sugar (10% solution) soaked cotton. In addition, control mosquitoes were exposed to non-impregnated paper for 60 minutes. Mortality was determined 24 hours post exposure to insecticides with dead and alive mosquitoes separately kept on desiccant (silica gel) and stored in -80°C respectively for molecular analyses.

### Bio-efficacy of commercialized nets against malaria vectors in Obout

To evaluate the impact of resistance on insecticide-based interventions against malaria vectors in this region, and given the deficiency of *Anopheles gambiae*, we checked the efficacy of common bed nets recommended by WHO against the Obout *An*. *funestus* population. Three minutes cone bioassays were carried out following the WHO guidelines [18]. Five batches of ten F_1_ females (2–5 days old) were placed in plastic cones attached to 5 commercial nets: PermaNet 3.0 (side of the net; deltamethrin 2.8g/kg) (Vestergaard, Lausanne, Switzerland), PermaNet 3.0 (top of the net; deltamethrin 4.0 g/kg plus 25g/kg piperonyl butoxide (PBO)) (Vestergaard, Lausanne, Switzerland), Olyset (2% permethrin) (Sumitomo Chemical UK PLC, London, UK) and Olyset plus (2% permethrin plus 1% PBO) (Sumitomo Chemical UK PLC, London, UK).

To evaluate the sub-lethal effect of PermaNet 2.0 (deltamethrin 1.8 g/kg) (Vestergaard, Lausanne, Switzerland) and the impact of exposure to this net on the life traits of Obout *Anopheles* population, a test was carried out for this net (in three replicates) on the populations of *An*. *funestus* of the first generation and *An*. *gambiae* of the seventh generation. This assay was performed repeatedly until 420 alive after exposure (resistant) and 420 controls (not exposed to insecticides) were obtained. After a threeminute exposure, the mosquitoes were transferred to paper cups containing 10% sugar solution. Mosquitoes alive post exposure were kept in the insectary to assess the effects of exposure on different life traits of the vectors.

### Evaluating the effect of PermaNet 2.0 on longevity of the vectors after exposure

300 alive females from each group (experimental and control) after exposure to PermaNet 2.0 were collected and split into 12 cups each. Dead mosquitoes were counted each morning and removed from the cups and those alive counted and fed with a 10% sucrose solution every day (from the beginning to the end of the experiment in each cup). The L119F mutation was genotyped in 25 females of the first generation (F_1_) at different days (2, 5, 10, 20) in the groups of females exposed and unexposed to PermaNet 2.0 to assess its association with a potential increased ability to survive after exposure to the insecticide.

### Assessing the effect of PermaNet 2.0 on the blood feeding ability

The remaining 120 females alive from each group (experimental and control) were divided into four cups each. 60 males of *An*. *gambiae* s.l. and from *An*. *funestus* s.l. respectively were introduced into each cup to increase the chances of mating. Three days later, the females were blood fed for 15 min during four consecutive days and the number of females successfully blood fed, were counted each day as well as the number of females fed and not fed by cup.

### Genotyping of resistance markers

The L119F-GSTe2 metabolic and A296S-RDL target-site resistance markers, involved in DDT/permethrin and dieldrin resistance in *An*. *funestus* respectively were firstly genotyped in the field collected *An*. *funestus* to assess the implication of these markers in the resistance observed in the locality. The L119F resistant marker was genotyped using the Allele-Specific PCR method recently designed [19] whereas the A296S was genotyped using a protocol previously described [7]. The L119F mutation and the L1014F associated with pyrethroids resistance in *An*. *gambiae* were genotyped also in mosquitoes alive after exposure to PermaNet 2.0 to assess the association between these mutations and the ability of mosquitoes to survive after exposure. The L1014F mutation was genotyped using a Taqman (Santa Clara, CA, USA) method as previously described [15, 16].

### Data analysis

The data were analyzed using "R" v. 3.4.0 software for Windows. Survival curves were estimated from the Kaplan-Meier estimator and compared by the non-parametric log rank test. The χ^2^ test was used to compare the proportions and the non-parametric U test of Mann-Whitney to compare the average number of eggs and larvae obtained. Association between L119F-GSTe2 genotypes and post-exposure longevity was estimated by calculating the odds ratio based on Fisher's exact probability test in the group of females exposed and the group of females unexposed to the net.

## Results

### Field collection and mosquito species identification

A total of 200 *An*. *funestus* s.l. females were collected in Obout during this study. Molecular identification of these mosquitoes by PCR [13] revealed that they all belong to *An*. *funestus* s.s. Molecular identification of the *An*. *gambiae* strain originally from Obout (seventh generation; F7) revealed 75.8% of *An*. *gambiae* s.s., 2.46% of *An*. *coluzzii* and 21.75% of hybrids *An*. *gambiae* / *An*. *coluzzii*.

### *Plasmodium* infection rate

**Fig 1:**
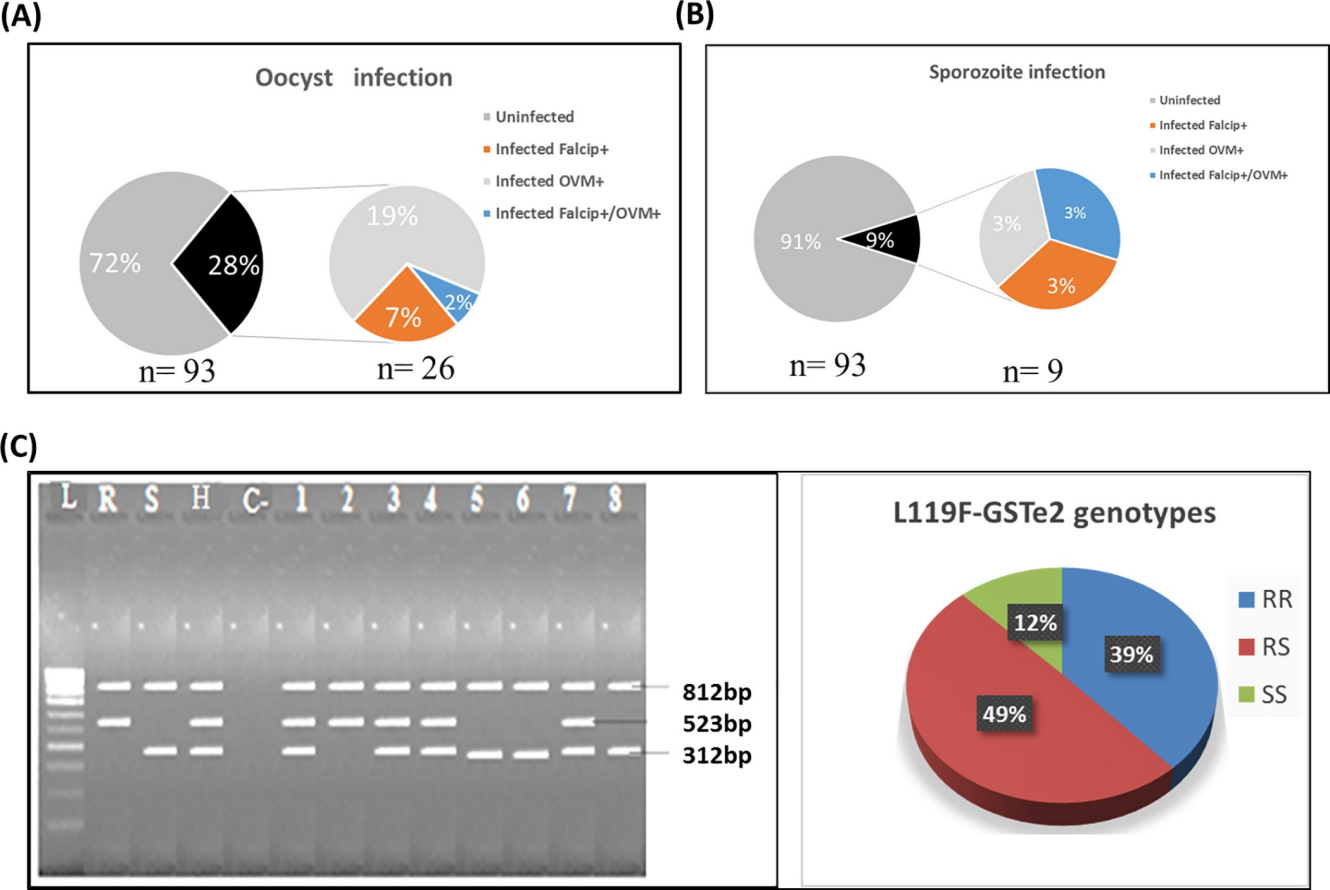
Characterisation of field-collected mosquitoes from Obout. *Plasmodium* infection status using abdomen (A) and head/thorax (B) of mosquitoes; (C) Gel picture showing the three genotypes at L119F locus for the *GSTe2* gene and the distribution of genotypes in Obout *An*. *funestus* population: L = ladder (100bp); Positive controls (R = homozygote resistant, S = susceptible, H = heterozygous; c- = negative control; 1, 3, 4, 7 = heterozygous individuals; 2 = resistant homozygous individual; 5, 6, 8 = susceptible homozygous individuals.

In total, 93 field-collected mosquitoes were dissected into head/thorax and abdomen for the detection of *Plasmodium* oocysts and sporozoites. TaqMan assays revealed that 27.9% (26/93) *An*. *funestus* mosquitoes (F_0_) were infected with *Plasmodium* oocysts including 6.4% (6/93) *P*. *falciparum* (falcip+), 19.3% (18/93) of *P*. *ovale/malaria/vivax* (OVM+) and 2.1% (2/93) mix infection with falcip+ and OVM+. At sporozoite stage, 3.2% (3/93) were infected with falcip+, 3.2% (3/93) with OVM+ and 3.2% (3/93) mix infection with falcip+ and OVM+ corresponding to overall sporozoite infection rate of 9.7% (9/93) (Fig 1A and 1B).

### Resistance profile of malaria vectors

**Fig 2:**
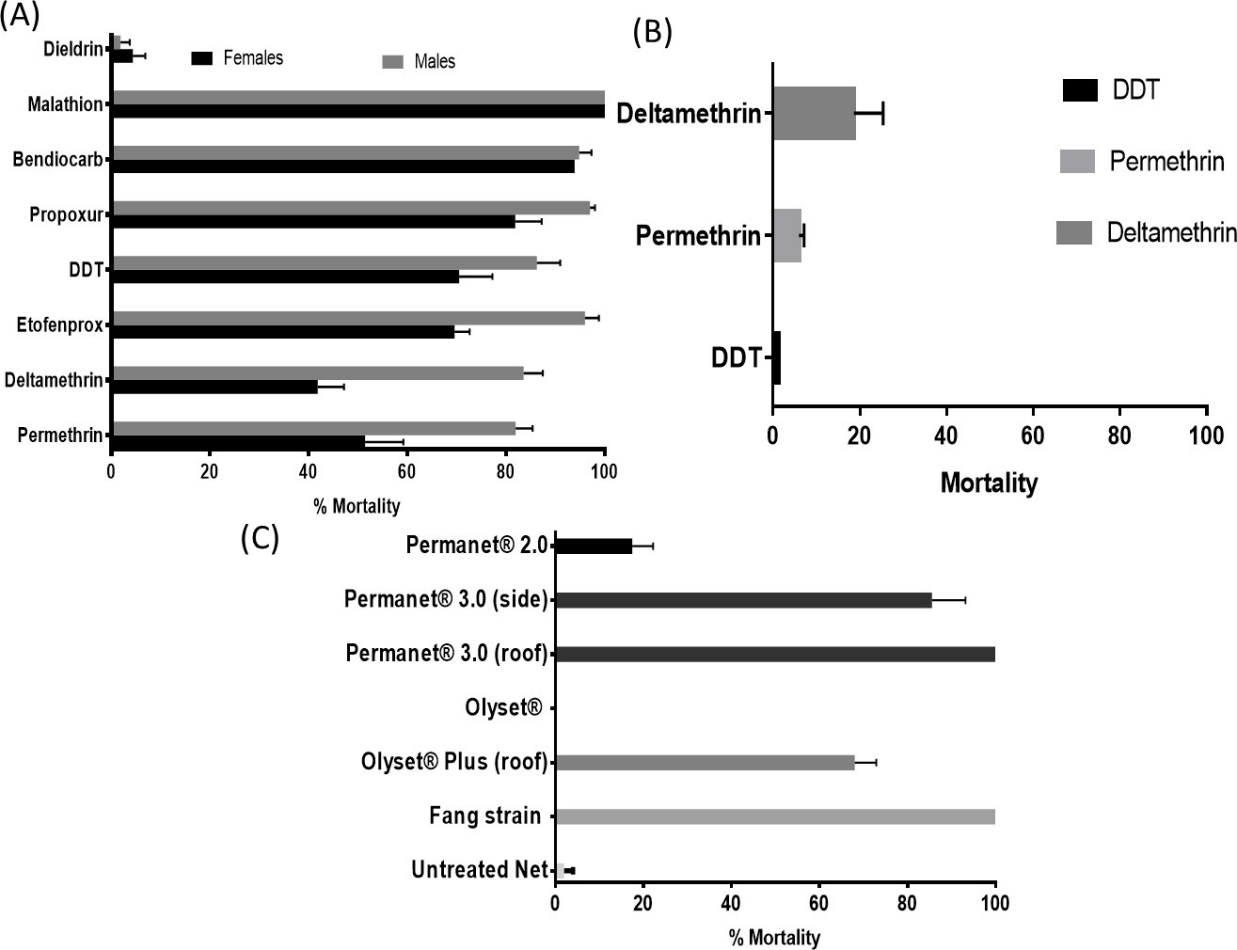
Resistance status of F_1_ from field-collected mosquitoes in Obout. **(A)** Susceptibility profile to World Health Organization insecticide susceptibility tube assays of *An*. *funestus* s.s (A) and *An*. *gambiae s*.*l* (B), (C) Bioefficacy of different commercial long-lasting insecticidal nets in *An*. *funestus* s.s. Error bars represent standard error of the mean (n = 4). Abbreviations: DDT, dichlorodiphenyltrichloroethane.

Around 1800 F_1_ adults were exposed to various insecticides. *An*. *funestus* of both sexes from Obout exhibited full susceptibility to the organophosphates, malathion and fenitrothion with 100% mortality rate observed 24 hours post exposure to both insecticides. Various levels of resistance to insecticides were noticed for other three classes of insecticides, with mosquitoes exhibiting very high resistance to dieldrin (mortality = 4.35±2.5%) (Fig 2A). Resistance was observed against the pyrethroids permethrin (type I; 51.4 ±7.8% mortality), deltamethrin (type II; 41.8 ±5.3% mortality), etofenprox (69.6± 3.1% mortality) and organochlorine DDT (70.5± 6.8% mortality). Only moderate resistance level was recorded with the carbamates bendiocarb (93.9± 1.1%) and propoxur (81.7± 5.3%). Bioassay conducted on *An*. *gambiae* F_1_ revealed higher resistance level including against DDT (mortality = 1.3%), to deltamethrin (mortality = 18.7%) and permethrin (mortality = 5.9%) (Fig 2B).

### Assessment of bed net efficacy using cone assays

A significant contrast in efficacy was observed between pyrethroid-only and PBO-based nets. A significantly low efficacy was observed for both pyrethroid-only nets with a complete loss observed for Olyset with 0% mortality whereas PermaNet 2.0 induced only 17.5% against *An*. *funestus* (Fig 2C). For this last net, a similar observation was made for *An*. *gambiae* (F_7_) from the same location with 16.4% mortality. On the contrary, PBO-based nets exhibited a significantly greater efficacy with the highest level observed with the top part PermaNet 3.0 with 100% mortality. Similarly, the side part of PermaNet 3.0 (85.6 ± 7.6%) and Olyset *Plus* (68.0 ±4.9%) also presented a high mortality rate. Susceptible strains for both species, FANG (*An*. *funestus*) and Kisumu (*An*. *gambiae*) showed a full susceptibility to all nets.

### Effect of PermaNet 2.0 on the blood feeding ability of mosquitoes

**Fig 3:**
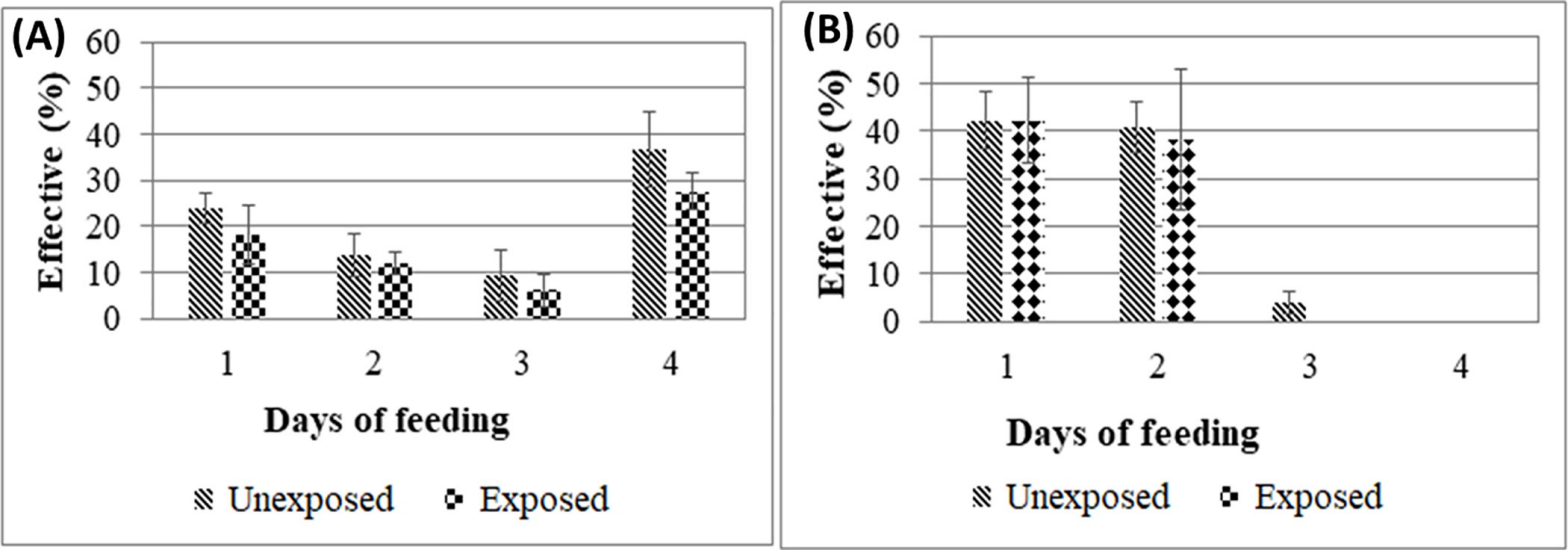
Impact of exposure to PermaNet 2.0 on the blood meal intake of *An*. *funestus* and *An*. *gambiae*. Comparison of the dynamic of blood feeding between mosquitoes exposed and unexposed to permaNet 2.0 in *An*. *gambiae* s.l. (A) and *An*. *funestus* s.l. (B).

To evaluate the effect of PermaNet 2.0 on females’ ability to take a blood meal, a study was conducted for 4 days for 15 minutes on 120 females of each group (Fig 3). Over the 4 days of blood feeding, no significant difference was found in the blood feeding ability of unexposed *An*. *gambiae* compared to those exposed to the net (*p* = 0.3) (Fig 3A). However, when the proportion of females fed every day were compared, no significant difference was observed between the two groups (χ2 = 0.23, *p* = 0.97). In contrast to *An*. *gambiae*, females *An*. *funestus* took blood over three days for the unexposed and two days for those exposed (Fig 3B). The daily comparison of the proportions of females fed, revealed that there is no significant difference between the two groups (χ2 = 3.99, *p* = 0.14). This result suggests that PermaNet 2.0 had no effect on female blood sampling in *An*. *funestus* s.s.

### Effect of PermaNet 2.0 on the longevity of mosquitoes

To evaluate the effect of PermaNet 2.0 on the longevity of the malaria vectors after exposure to the net, we used a Kaplan-Meier survival estimator to estimate the survival functions; followed by the non-parametric test of log-rank, to compare the survival curves. It was noted that the maximum life time was 20 days for *An*. *gambiae* s.l. and 30 days for *An*. *funestus* s.s.

**Fig 4:**
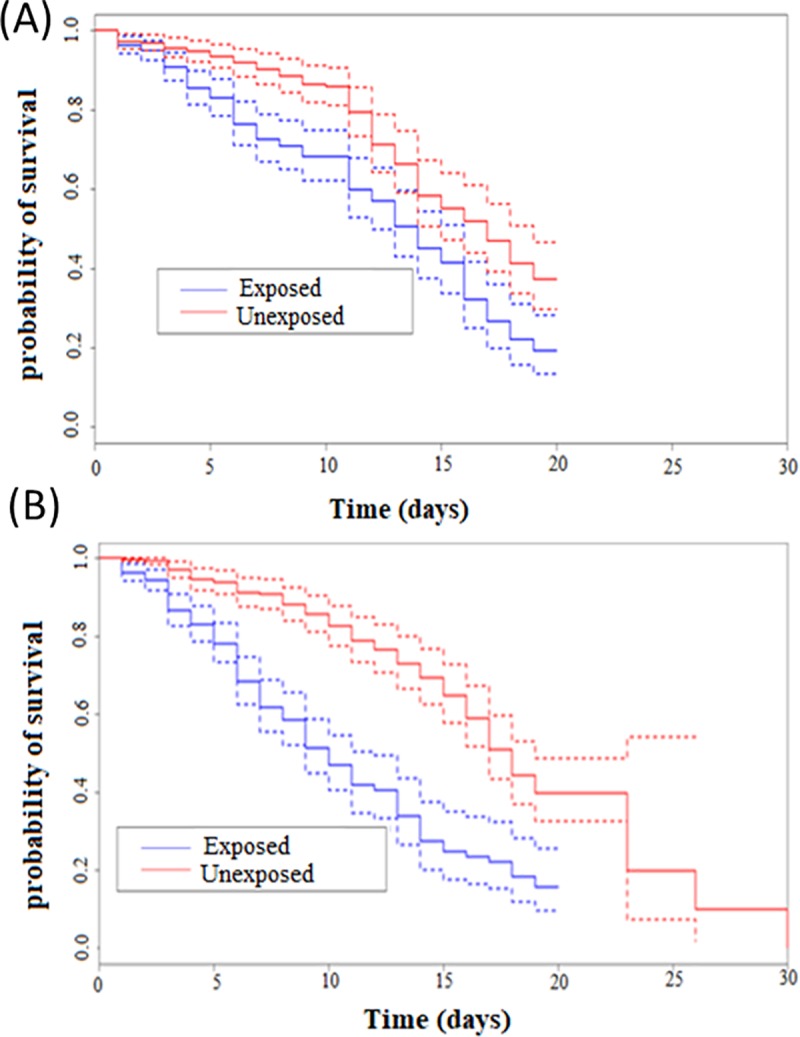
Impact of exposure on the longevity of the vectors. Kaplan-Meier survival curves of *Anopheles gambiae* s.l. (A) and *Anopheles funestus* s.s. (B) after exposure to PermaNet 2.0. Blue lines indicate mosquitoes exposed to the net while red lines indicate mosquitoes unexposed.

Comparison of survival curves in *An*. *gambiae* exposed to the net (Fig 4A, line in blue) and unexposed (Fig 4A, line in red). Showed that, there is a variation of the two curves over time. The log-rank test used revealed that females unexposed to the net lived significantly longer (average life = 11.25 ± 1.12) than exposed females (average life = 8.87 ± 1), (χ2 = 20, *p* <0.001); showing that although a reduced efficacy of this net against resistant mosquitoes, there is a long-term effect on *An*. *gambiae*.

In *An*. *funestus*, comparison of survival curves between mosquitoes exposed (Fig 4B, line in blue) and those unexposed (Fig 4B, line in red) to the net showed considerable difference between both groups towards the third day. The log-rank test used revealed that unexposed females lived significantly longer (average life = 12.46 ± 1.16) than those exposed (average life = 6.95 ± 0.73), (χ2 = 62.8, *p* <0.001). This result highlights the same negative impact of PermaNet 2.0 exposure on longevity in *An*. *funestus* as in *An*. *gambiae*.

### Genotyping of L119F-GSTe2 metabolic and A296S-RDL target site resistance in *An*. *funestus s*.*s* field population

Genotyping of L119F-GSTe2 mutation in indoor collected females revealed a frequency of 63.2% for the 119F resistant allele comprising 38.5% (35/91) 119F/F-RR homozygous resistant, 49.4% (45/91) 119L/F-RS heterozygotes and 12.1% (11/91) L/L119-SS homozygous susceptible (Fig 1C). The A296S-RDL mutation was tested in 100 field-collected. Out of these mosquitoes tested, 97 were homozygote resistant and 3 heterozygous resistant genes corresponding to the frequency of almost 100% for the 296S resistant allele.

### Assessment of association between the L119F mutation and longevity after exposition to PermaNet 2.0

**Fig 5:**
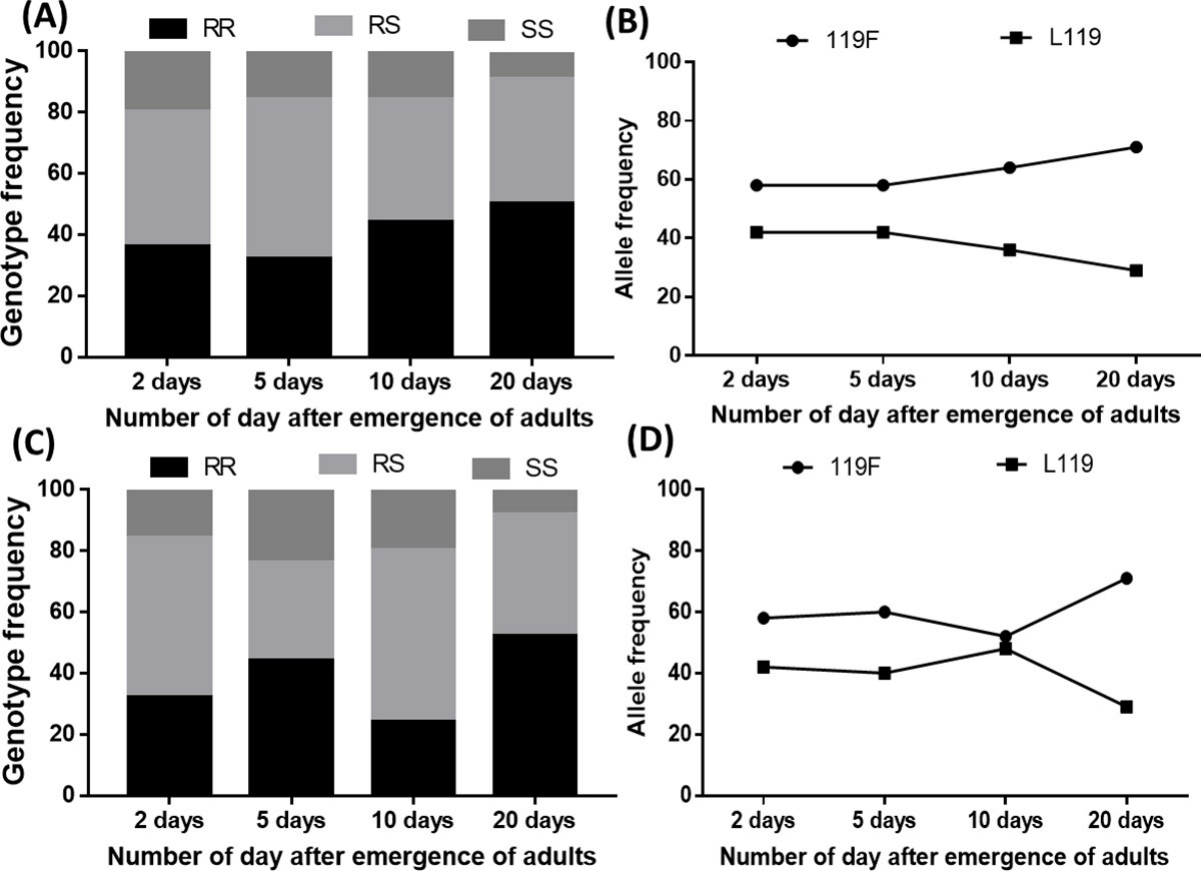
Influence of L119F-GSTe2 mutation on the adult longevity of *An*. *funestus*. Distribution of L119F genotypes (**A**) and alleles (**B**) in mosquitoes exposed to the net at different time in the survived mosquitoes; (**C**) and (**D**) distribution of genotypes and alleles respectively in unexposed mosquitoes at different time point after the emergence of adults.

A total of 200 mosquitoes were genotyped for this mutation. All three genotypes were successfully detected by the newly designed AS-PCR method (Fig 1C). In the group of mosquitoes exposed to the net and those unexposed, 25 mosquitoes were genotyped in days 2, 5, 10 and 20 post exposure. Overall, the genotypes were equally distributed in both group (x^2^ = 0.76, *p* = 0.7) with the predominance of heterozygous L119F-RS (44.8%) followed by homozygous resistant 119F/F (39.1%) and then susceptible homozygous L/L119 (16.1%) in the group of mosquitoes alive after exposed to the net and 39% homozygous resistant 119F/F, 44% heterozygous L119F-RS and 17% homozygous susceptible L/L119 in the group of unexposed mosquitoes. A non-significant increased proportion of homozygote resistant mosquitoes coupled with increased frequency of 119F resistant allele was observed in both group from D2 to D20 (x^2^ = 2.04, *p* = 0.9) (Fig 5A & 5B) although a reduction of this proportion was noticed in D10 in the group of mosquitoes unexposed to the net (Fig 5C & 5D).

The evaluation of the association of each genotype with longevity was performed by estimating the ratio of odds between genotypes in each sample group (Table 1). In both group, mosquitoes with RR genotype displayed a significant greater longevity compared to other genotypes. In the group of mosquitoes exposed to the net, the ability of RR to survive was higher but not significant compared to RS from D2 to D20 (OR = 1.4 CI at 95%: 0.8–2.7 *p* = 0.28) but significant when compared to SS (OR = 3.5 CI at 95%: 1.4–8.7 *p* = 0.01). In addition, heterozygote mosquitoes displayed also a significantly greater longevity compared to SS (OR = 2.4 CI at 95%: 0.9–6 *p* = 0.04). In the group of mosquitoes exposed to untreated net the pattern was almost the same, but the ability of RR was significantly greater than RS (OR = 2.1 CI at 95%: 1.1–3.9 *p* = 0.02) (Table 1). The effect of the mutation is greater for homozygous resistant individuals (RR) as they have a higher longevity than the heterozygotes (RS) suggesting an additive impact of the resistant allele on the longevity of mosquitoes. Furthermore, evaluation of the association of each genotype with survival in exposed females compared to unexposed ones (Table 2) revealed that the ability of RS mosquitoes to survive is lower in the 5^th^ and 10^th^ days post exposition compared to both homozygote resistant (OR = 2.2 CI at 95%: 1.18–4.20; *p* = 0.01) and susceptible (OR = 0.41 CI at 95%: 0.19–0.89; *p* = 0,035) suggesting a potential heterozygote disadvantage.

**Table 1:** Evaluation of the association between different genotypes/alleles of the L119F-GSTe2 mutation and female longevity during observation period of 20 day in mosquitoes exposed and unexposed to the net.

Genotypes	D_2 ₓ_ D_5_		D_2 ₓ_ D_10_		D_2 ₓ_ D_20_		D_5 ₓ_ D_10_		D_5 ₓ_ D_20_		D_10 ₓ_D_20_	
	OR	*p-*value	OR	*p-*value	OR	*p-*value	OR	*p-*value	OR	*p-*value	OR	*p-*value
**Exposed mosquitoes**												
**RR vs RS**	0.75	0.45	1,34	0.43	1.46	0.28	1.93	0.04 ^S^	1.93	0.04 ^S^	1.08	0.92
	(0.40–1.40)		(0.73–2.49)		(0.80–2,66)		(1.06–3.53)		(1.06–3.53)		(0,60–1,96)	
**RR vs SS**	1,11		1,52		3.47	0,01^S^	1.56	0.39	3.13	0.03 ^S^	2.27	0.13
	(0,49–2,50)	1.00	(0.69–3.37)	0.39	(1.38–8.76)		(0.69–3.56)		(1,20–8,14)		(0.89–5.82)	
**RS vs SS**	1.48	0.42	1,14	0.92	2.38	0.10	0.77	0.66	1.62	0.44	2.10	0.19
	(0.68–3.19)		(0.52–2.49)		(0,95–6.00)		0.34–1.72)		(0.63–4.14)		(0.81–5.45)	
**R Vs S**	1.00	0.56	1.29	0.23	1.77	0.04 ^S^	1.29	0,23	1.78	0.04 ^S^	1.38	0.18
	(0.57–1.75)		(0.72–2.28)		(0.99–3.19)		(0.73–2.78)		(0.99–3.19)		(0.76–2.50)	
**Unexposed mosquitoes**												
**RR vs RS**	2.23	0.02 ^S^	0.88	0.81	2.11	0.02 ^S^	0.39	0.007 ^S^	0.95	1.00	2.41	0.008 ^S^
	(1.19–4.21)		(0.46–1.65)		(1.15–3.86)		(0.21–0.75)		(0.51–1.75)		(1.30–4.76)	
**RR vs SS**	0.92		0.70		3.25	0.02 ^S^	0.76	0,61	3.55	0.008 ^S^	4.64	0.002 ^S^
	(0.42–2.00)	1.00	(0.31–1.61)	0.53	(1.25–8.46)		(0.36–1.63)		(1.44–8.68)		(1.81–11.88)	
**RS vs SS**	0.41	0,04 ^S^	0.80	0.70	1.54	0,51	1.95	0,11	3.75	0.008 ^S^	1.92	0.23
	(0.19–0.89)		(0.37–1.71)		(0.60–3.95)		(0.93–4.08)		(1.49–9.46)		(0.77–4.81)	
	1.1	0.44	0.85	0.33	1.86	0.02 ^S^	0.78	0.24	1.71	0.05 ^S^	2.19	0.006 ^S^
**R Vs S**	(0.61–1.91)		(0.46–1.49)		(1.03–3.56)		(0.54–1.37)		(0.94–3.10)		(1.22–3.94)	

Abbreviations: D = day; RR = homozygous resistant; RS = heterozygous resistant; SS = homozygous Susceptible; s = significant for the OR the 95% confidence interval are indicated in the brackets

**Table 2:** Evaluation of the association between the L119F mutation and survival ability in exposed mosquitoes compared to unexposed group.

	D2		D5		D10		D20	
Genotypes	OR (95% CI)	P- value	OR (95%CI)	P- value	OR (95%CI)	P- value	OR (95%CI)	P- value
RR vs RS	0.75 (0.40–1.40)	0.45	2.23 (1.18–4.20)	0.01S	0.50 (0.26–0.91)	0.04S	1.08 (0.54–1.90)	0.92
RR vs SS	1.11 (0.42–2.50)	1.00	0.91 (0.19–4.35)	1.00	0.50 (0.22–1.14)	0.15	1.08 (0.60–1.93)	0.92
RS vs SS	1.47 (0.68–3.19)	0.92	0.41 (0.19–0.89)	0.035S	1.04 (0.48–2.26)	0.92	0.92 (0.40–2.12)	1.00
R vs S	1.00 (0.57–1.75)	0.56	1.09 (0.62–1.90)	0.44	0.66 (0.37–1.16)	0.10	1.05 (0.57–1.94)	0.50

Abbreviations: D = day; RR = homozygous resistant; RS = heterozygous resistant; SS = homozygous Susceptible;s = significant for the OR the 95% confidence interval are indicated in the brackets

## Discussion

Insecticide-resistance constitutes a serious threat to malaria control programs because it can reduce the efficacy of control vector tools and therefore potentially jeopardizing malaria control. However, the impact of insecticide-resistance on malaria transmission remains challenging to establish despite the threat it poses to malaria prevention. By evaluating the effect of LLIN exposure on the life traits of major malaria vectors, this study has contributed to improve our understanding of the long-term impact of LLINs on these vectors in the context of pyrethroid resistance.

### Species composition of malaria vectors in the study site

Only *An*. *funestus* s.l. was collected in Obout during this study. This can be linked to the fact that collection was done during the dry season. The presence of *An*. *funestus* s.s. only from the *An*. *funestus* group further indicated that only this species is extremely anthropophilic and endophilic in the group) and would be the most involved in the transmission of human malaria [20]. These results further support the widespread geographical distribution of *An*. *funestus* in Cameroon where it is a major malaria vector [21, 22].

Identification of the species belonging to the *An*. *gambiae* complex using the mosquitoes maintained in lab condition for seven generation revealed the dominance of *An*. *gambiae* s.s. at a frequency of 75.8% against 21.75% hybrids and 2.46% of *An*. *coluzzii*. The dominance of *An*. *gambiae* s.s. compared to other species from *An*. *gambiae* complex supports the work carried out in Cameroon which mentioned the predominance of this species in rural areas [23–25]. The high proportion of hybrids observed would be due to a laboratory confinement which can increase the chance of mating of both species [26] given that the tested individuals were kept in the insectary conditions for seven generations. However, this result differs from field observations in Cameroon where there is an absence of hybrids in natural field populations [23, 27]. However, this observation corroborates with observations made in West Africa (Guinea Bissau) where higher hybridization rates (from 19% to 24%) are generally observed between these two species [28, 29] compared to Central Africa. This result suggests that reproductive isolation between the two species is not complete.

### Resistance of the vectors to pyrethroids undermines vector control strategies

Profiles of insecticide resistance of the *An*. *funestus* and *An*. *gambiae* s.l. Obout populations is similar to patterns of resistance previously been reported in Cameroon for these species with resistance against all pyrethroids and full susceptibility to organophosphates [30–32]. Resistance to pyrethroids in both species is associated with a significant decrease of efficacy of all pyrethroid-only LLINs as shown by the very low mortality rates against PermaNet 2.0 (<20%) for both species and the no mortality at all in the case of Olyset net. Such loss of efficacy of these pyrethroid-only nets is similar to cases reported in other locations in Cameroon [33] and Africa [34, 35] decrease in the efficiency of PermaNet 2.0 at a mortality rate of 22% in the zone of high resistance has been described in *An*. *gambiae* s.s.. In contrast, PBO-based nets exhibited a greater efficacy with the highest presented by PermaNet 3.0 top with 100% mortality suggesting that cytochrome P450 genes probably are the main genes driving pyrethroid resistance in this location. This higher efficacy of PBO-based nets is similar to results reported for *An*. *funestus* in other locations including Cameroon [33], Malawi [7], DR Congo [35]. Such results suggest that PBO-based nets should be considered as an alternative to pyrethroid-only nets in areas of high resistance driven by metabolic resistance mechanisms notably cytochrome P450s as it is the case for *An*. *funestus* [36].

### Exposure to PermaNet 2.0 negatively influences the longevity of malaria vectors

Vector longevity is one of the most important biological traits that ensures the complete development of *Plasmodium* until it is transmitted to the host. Evaluating the effect of PermaNet 2.0 on the longevity of *An*. *funestus* s.l. and *An*. *gambiae* s.l. revealed a significant decrease in the life span of both vectors after exposure to the net. Mean life expectancy was approximately reduced by 2 days in *An*. *gambiae* s.l. and 5 days in *An*. *funestus* s.l. This reduction could be an epidemiological barrier to *Plasmodium* transmission because the vectors have to live sufficiently longer to ensure the parasite transmission [37]. Indeed, at a temperature of about 25°C, *P*. *falciparum* needs 10 to 12 days to complete its cycle [38]. However, exposed females had an average life-span of less than 10 days. In insects, variation in longevity may be a consequence of energy loss and oxidative stress [39]. The decrease in survival observed in exposed females may be associated with either lower energy consumption due to shock produced during exposure that would be beneficial for survival, or neurodegeneration that may lead to apoptosis. It can also be attributed to a high level of oxidative stress because the unbalanced production of reactive oxygen species (ROS) is detrimental according to age-free radical theory [40]. These observations are similar to those made on the resistant laboratory strains of *An*. *gambiae* s.l. (the TIA strain from southern Côte d'Ivoire and the TOR strain from Uganda) in contact with PermaNet 2.0 [8]. This suggests that a non-lethal effect observed 24h post-exposure to the net is not enough to conclude on the impact of insecticide resistance on effectiveness of control measures. Also, LLINs remain a barrier to mosquito biting and resistant mosquitoes can still be repelled by insecticides (Sharma et al., 2005), thus control interventions still confer some personal protection to the user. However, it is always preferable to use nets that can kill mosquitoes immediately. The new generation nets such as Permanet 3.0 or Olyset Plus could be the alternative measures as they contain the PBO which increases the efficacy of the nets but this is efficient only in situations where the resistance is driven by Cytochrome P450 and these nets are not accessible to everybody because of the cost.

### No effect of PermaNet 2.0 exposure on the blood feeding ability of mosquitoes

Evaluation of the effect of PermaNet 2.0 on the blood feeding ability of mosquitoes revealed that this net would have no effect on the vector's blood meal intake. In practice, *An*. *funestus* s.l. and *An*. *gambiae* s.l. generally require a second blood meal a day after the first [41] to ensure maturation and egg laying. However, during the experiment, *An*. *gambiae* s.l. fed at different frequencies over its duration with a major peak on the fourth day (last day of the experiment). The peak observed is due to an inhibition of the usual feeding behavior (search for the blood meal) during the 40 hours following a blood supply in a laboratory condition, accompanied by an inhibition of the olfactory faculties with regard to certain substances (perspiration, indole, etc.). This eating behavior is restored 72 hours after a blood meal [42]. However, studies using experimental huts could provide more information about the effect of this net on the blood meal intake as this is closer to the natural condition.

### Impact of the L119F-GSTe2 mutation on the longevity of *Anopheles*

The 119F-GSTe2 resistance allele associated with DDT/Pyrethroids resistance was detected with high frequency (61%) in this locality correlating with high pyrethroid/DDT resistance levels. This high frequency of 119F-GSTe2 is probably associated to the massive use of LLINs and/or agricultural pesticides in the locality. This result is similar to those recently conducted in Africa, particularly in northern Cameroon [43] and Benin in Pahou and Kpome [44], which highlighted the presence of the 119F-GSTe2 allele at a high frequency of 52% and 45% respectively. In this study, in the group of mosquitoes exposed to treated and untreated net, mosquitoes with RR genotype showed a significant increase longevity compared to those with RS and SS genotypes. In addition, RS mosquitoes had also a greater longevity compared to SS suggesting that possessing the 119F resistant allele increases the ability of mosquitoes to survive after exposition to the insecticide. The increased longevity in GSTe2-resistant mosquitoes could be associated to the implication of GSTe2 in oxidative stress as noticed in other studies [45] [46, 47]. In fact, it was mentioned that GSTs protect mosquitoes against oxidative stress which results in the increase longevity of the vector [48]. The increased longevity in resistant mosquitoes observed here is likely to increase the vectorial capacity of resistant mosquitoes in the field even in the presence of bed nets as female mosquitoes must live sufficiently longer to be able to transmit the parasite. The increased longevity in resistant mosquitoes even in the group of mosquitoes exposed to untreated net is confirmed by the study of Tchouakui et al [45] where it was noticed during an observation period of 30 days in the absence of insecticide that mosquitoes with RR genotype for *GSTe2* had a significantly increased longevity compared to other genotypes. This observation confirmed the role of *GSTe2* in increasing the longevity of the vector which is problematic for control programs as this might increase the transmission risk in the field population. Nevertheless, a reduced proportion of resistant mosquitoes was observed in D10 as noted by Tchouakui *et al*. also. Even if this difference was not significant probably due to low sample size, further studies are needed to evaluate the effect of other factors on the GSTe2 expression at this time point.

## Conclusion

These results show that although a conventional net such as PermaNet2.0, presents a reduced efficacy against pyrethroid resistant population, it remains efficient after exposure by reducing the life expectancy of the vectors which could contribute in the reduction of malaria incidence.
